# Editorial: Livestock and poultry infectious diseases: pathogenesis and immune mechanisms

**DOI:** 10.3389/fcimb.2023.1249034

**Published:** 2023-07-11

**Authors:** Jiaqiang Wu, Jiabo Ding, Leyi Wang

**Affiliations:** ^1^Shandong Key Laboratory of Animal Disease Control and Breeding, Institute of Animal Science and Veterinary Medicine, Shandong Academy of Agricultural Sciences, Jinan, China; ^2^Institute of Animal Sciences, Chinese Academy of Agricultural Sciences, Beijing, China; ^3^Veterinary Diagnostic Laboratory, Department of Veterinary Clinical Medicine, College of Veterinary Medicine, University of Illinois, Urbana, IL, United States

**Keywords:** livestock, poultry, infectious diseases, pathogenesis, immune mechanisms

Livestock and poultry infectious diseases cause substantial losses to the relevant industries, and even some are zoonotic diseases posing great threat on public health. At present, the epidemic modes of livestock and poultry infectious diseases are becoming more and more complex, and the new diseases are constantly emerging, thus raising standards for the prevention and control of these diseases. This Research Topic aims to highlight the recent research about pathogenesis and immune mechanisms of livestock and poultry diseases, which may contribute to prevention and control of these diseases.

This Research Topic collects the latest discoveries to understand the molecular pathogenesis of livestock and poultry diseases, host immune responses to diseases, and advanced methods to better detect and control these diseases. Among 11 submissions, seven were published in this Research Topic, including three original research papers on the pathogenesis of extraintestinal pathogenic *Escherichia coli* (ExPEC), *Salmonella Typhimurium* and Chicken infectious anemia (CIA), one original research paper on the antiviral mechanisms of a new drug, one original research paper on the innate immune responses of neonatal calves to parasites, one original research paper on construction of a new recombinant adenovirus and one brief research report on a new detect methods of antibodies specific to African swine fever virus (ASFV).

ExPEC is an important zoonotic pathogen, causing infections of urinary tract, bloodstream, prostate, and other infections at non-intestinal sites (Sora et al., 2021). Although numerous virulence factors have been identified in diverse ExPEC pathotypes, the underlying mechanisms of sepsis caused by ExPEC remains to be explored. Pan et al. reported a high virulent ExPEC strain PU-1, which has a robust ability to colonize within host bloodstream, while induced a low level of leukocytic activation. This study found that the two serine protease autotransporters of Enterobacteriaceae (Vat^PU-1^ and Tsh^PU-1^) play critical roles to cause a heavy bacterial load within bloodstream via immunomodulation of leukocytes, thus providing a more comprehensive understanding about how ExPEC colonizes within host bloodstream and causes severe sepsis.

*S. Typhimurium* is the leading cause of foodborne illnesses, resulting in major epidemics and economic losses in recent years (Galán, 2021; Aguilera et al., 2022). Guo et al. constructed successfully a *galU* gene mutant of *S. Typhimurium* by red homologous recombination technology and studied its biological characteristics. This study demonstrated *GalU* plays an important role in autoagglutination, biofilm formation, antibiotic sensitivity, serum and egg albumen sensitivity, cell adhesion ability, and pathogenicity in *S. Typhimurium* from chicken, which revealed that *galU* is an important virulence factor in the pathogenicity of *S. Typhimurium*. *galU* may serve a target for the development of veterinary drugs for the prevention and control of *S. Typhimurium*.

CIA is an immunosuppressive disease caused by chicken anemia virus (CAV), which invades bone marrow hematopoietic cells and T lymphocytoblasts of immune organs (e.g., thymus) in young chickens, leading to increased mortality due to secondary complications. Fang et al. isolated a highly pathogenic CAV strain SD15 from a two-month-old chicken with severe anemia and analyzed the genetic evolution relationship. They found some genetic characteristics of the highly pathogenic novel CAV strain that distinguish it from other CAV strains, which provided a better understanding of the critical factors determining the pathogenicity of CAV strains. The challenge test of SD15 strain implied that the pathogenicity of epidemic CAV strains might increase, and that CAV has the potential ability to break age resistance. Overall, these findings may contribute to better prevention and treatment of CAV infections.

Porcine epidemic diarrhea virus (PEDV) has caused significant economic losses to the swine industry worldwide (Jung et al., 2020). Although there are several treatment methods at present, there is still a lack of clinically effective targeted drugs. Zhang et al. established a model of erastin versus ferrostatin-1 treatment of Vero cells and studied the effect of erastin on the process of PEDV infection in Vero cells. Erastin can activate reactive oxygen species and lipid oxidation in Vero cells and inhibit the replication of PEDV through the regulation of ferroptosis pathway. This result suggested erastin may be a potential drug for the treatment of PEDV infection.

*Cryptosporidium parvum* is a zoonotic agent causing gastroenteritis in a diversity of vertebrates, including cattle, and it is a leading global cause of diarrhea, illness, and death in young children (Collaborators, 2017). So far, there are very limited prevention methods. Gamsjäger et al. used an experimental model of *C. parvum* challenge in neonatal calves and the results indicated that *C. parvum* infection in neonatal calves provoked severe diarrheic neutrophilic enterocolitis, perhaps augmented due to the lack of fully developed innate gut defenses. Colostrum supplementation showed limited effect on mitigating diarrhea but demonstrated some clinical alleviation and specific modulatory influence on host gut immune responses and concomitant microbiota.

Adenoviruses, belonging to the family *Adenoviridae*, have caused a variety of diseases in poultry and humans. Recently, the outbreaks of duck adenovirus 3 (DAdV-3) and highly pathogenic serotype 4 fowl adenovirus (FAdV-4) have posed a great threat to the duck industry in China (Yin et al., 2022). Guo et al. constructed a novel recombinant virus rFAdV-4-Fiber-2/DAdV-3 expressing the Fiber-2 protein of DAdV-3 using CRISPR/Cas9 and Cre-LoxP systems. The recombinant virus can efficiently replicate in LMH cells with a high yield, highlighting the value of the recombinant virus as a potential vaccine candidate against both FAdV-4 and DAdV-3.

African swine fever (ASF) is an acute, febrile and highly contagious infectious disease that causes severe economic losses to the global porcine industry. To date, no vaccines or effective drugs can be used for the prevention and control of ASF, so early diagnosis is the primary measure of preventing and controlling ASF. Shen et al. used the truncated ASFV I329L protein to develop an indirect enzyme-linked immunosorbent assay (iELISA) for ASFV antibody. The iELISA had good specificity and excellent repeatability and reproducibility, with comparable sensitivity to the indirect ELISA method described in previous studies. Therefore, it is well suited for the antibody detection against ASFV and epidemiological surveillance.

Finally, we thank all the authors who contributed their original work to our Research Topic and the reviewers for their valuable comments. We would like to express our sincere gratitude to the editorial office of Frontiers in Cellular and Infection Microbiology, for their excellent support and for providing us with this opportunity to hold this Research Topic successfully.

## Author contributions

JW drafted the manuscript. JD and LW revised it. All authors made a direct and intellectual contribution to the work and approved the final version for publication.
